# Serie de pacientes operados de masas y quistes cardíacos en un hospital general de España: Una experiencia de 20 años

**DOI:** 10.23938/ASSN.1060

**Published:** 2023-12-26

**Authors:** Adrián Gil-Korilis, Claudia Esquíroz-Patiño, Aroa Llamas-Llamazares, Rebeca Manrique, Agnes Diaz-Dorronsoro

**Affiliations:** 1 Universidad de Navarra Universidad de Navarra Facultad de Medicina Pamplona Navarra Spain; 2 Clínica Universidad de Navarra Departamento de Cardiología y Cirugía Cardiaca Pamplona Navarra Spain

**Keywords:** Cardiología, Cirugía Cardíaca, Enfermedades Cardiovasculares, Neoplasias Cardíacas, Mixoma, Cardiology, Cardiac Surgery, Cardiovascular Diseases, Heart Neoplasms, Myxoma

## Abstract

**Fundamento:**

Las masas y quistes cardíacos son entidades bien conocidas, cuya reducida prevalencia y sintomatología inespecífica dificultan su diagnóstico. El objetivo del estudio fue caracterizar el cuadro de los pacientes afectos en nuestro medio para orientar futuros diagnósticos.

**Metodología:**

Estudio descriptivo de los pacientes intervenidos de tumores y quistes cardíacos entre 2002 y 2022 mediante la búsqueda en el registro del Servicio de Cardiología y Cirugía Cardíaca de la Clínica Universidad de Navarra (Pamplona, España). Se recogieron variables sociodemográficas, clínicas, histológicas y quirúrgicas.

**Resultados:**

Se identificaron 13 pacientes, la mayoría (76,92%) mujeres, con media de edad 63,08 años (DE: 15,17). El 92,31% de los pacientes tenían al menos un factor de riesgo cardiovascular, siendo los más prevalentes un IMC ≥25 kg/m^2^ y la hipertensión arterial (61,54% y 53,85%, respectivamente). El tipo de masa cardíaca más frecuente fue el mixoma (69,23%). El 46,15% de masas cardiacas fueron hallazgos incidentales; el síntoma más frecuente fue la disnea (53,85%) y el 30,77% de los pacientes se encontraban asintomáticos. . La prueba de imagen más empleada para en el diagnóstico fue la ecocardiografía transtorácica Doppler color (69,23%). La concordancia entre los diámetros medios precirugía y postcirugía resultó muy alta (CCI = 0,807, IC95%: 0,450-0,943).

**Conclusiones:**

Se describieron los cuadros de los pacientes, aportando información poco descrita en la literatura, como los factores de riesgo cardiovascular más frecuentes en estas entidades. Se describieron un caso de leiomiosarcoma cardíaco y un caso de sarcoma intimal del tronco pulmonar, dos tipos de tumores extremadamente raros de los que existen pocos casos descritos.

## Abstract (en)

**Background:**

Masses and cysts in the heart are well-known entities, but their low prevalence and non-specific symptoms makes the diagnosis difficult. We aimed to characterize the features of these entities in our environment.

**Methods:**

We carried out a search of patients who underwent surgery for tumors and cysts in the heart between 2002 and 2022 in the registry of the Department of Cardiology and Cardiac Surgery of Clínica Universidad de Navarra (Pamplona, Spain). Sociodemographic, clinical, histological, and surgical variables were collected.

**Results:**

We identified 13 patients; mean age was 63.08 ± 15.17 years, 76.92% were female and 92.31% had at least one cardiovascular risk factor, e.g., BMI ≥ 25 kg/m2 and high blood pressure (61.54% and 53.85%, respectively). The most common type of cardiac tumors were myxomas (69.23%). Around half (46.15%) were incidental; the most frequent symptom was dyspnea (53.85%); 30.77% of the patients were asymptomatic. The most commonly used imaging technique for the diagnosis was transthoracic Doppler echocardiography (69.23%). The agreement between the mean diameters before and after surgery was very high (ICC = 0.807, 95%CI: 0.450-0.943).

**Conclusions:**

We describe the features of masses and cysts in the heart (77% female patients) and provide information scarcely available in the literature, e.g., the most frequent cardiovascular risk factors for this population. A case of cardiac leiomyosarcoma and a case of intimal sarcoma of the pulmonary trunk are described, two extremely rare tumors for which there are few described cases.

## INTRODUCCIÓN

Aunque bien conocidos por la comunidad sanitaria, la prevalencia de los tumores cardíacos es inferior al 0,3%^1^, con una incidencia acumulada de 13,8 casos por millón de habitantes al año en el caso de los tumores primarios^2^. El diagnóstico diferencial de estas neoplasias debe realizarse con otras masas cardíacas, como vegetaciones, trombos o hipertrofia miocárdica^3^, e incluso con otras entidades como neoplasias vasculares o quistes intrapericárdicos.

Los tumores cardíacos pueden clasificarse en tumores primarios y secundarios (metástasis)^4^, siendo estos últimos hasta 132 veces más frecuentes que los primarios^5^. Asimismo, los tumores primarios pueden clasificarse en benignos y malignos^6^, siendo el 75% benignos^7^. La práctica totalidad de los tumores cardíacos primarios benignos en el adulto corresponde al mixoma, cuya localización anatómica más habitual es la aurícula izquierda^9^. El tumor primario benigno más común en niños es el rabdomioma y, del 25% maligno, los más comunes son los sarcomas y los linfomas^8^.

Las masas cardíacas aparecen principalmente entre los 30 y los 60 años^10^ y algunos, como los mixomas, son más frecuentes en mujeres^3^. La histopatología de las masas cardíacas es muy diversa. Por ejemplo, los mixomas son estructuras gelatinosas formadas por células fusiformes estrelladas o poligonales, rodeadas de un estroma rico en glucosaminoglucósidos. Estas células se disponen en anillos alrededor de capilares amorfos y la superficie del tumor está rodeada de endotelio^11^^,^^12^.

La presentación clínica de las masas cardíacas no viene determinada por su histopatología sino por su tamaño, localización anatómica y movilidad^7^^,^^11^. Aun así, los síntomas suelen ser inespecíficos (dolor torácico, síncope, soplos o arritmias)^3^, lo que dificulta el diagnóstico al solaparse con la presentación típica de otras enfermedades cardiovasculares. Sin embargo, una elevada proporción de pacientes se encuentran asintomáticos en el momento del diagnóstico, descubriéndose el tumor de manera incidental a través de pruebas de imagen realizadas por otra causa^3^. La mayor calidad y empleo de las técnicas de imagen cardíaca en general en los últimos años ha contribuido a este hecho^13^.

Las recomendaciones actuales establecen la ecocardiografía como la prueba de elección para el diagnóstico de masas cardíacas, pudiéndose completar el estudio con otras modalidades de imagen como la tomografía computarizada (TC) o la resonancia magnética nuclear (RMN)^8^. A pesar de la gran información que pueden aportar estas pruebas, el diagnóstico definitivo debe ser histológico^11^.

El tratamiento de las masas cardíacas se basa en su escisión quirúrgica independientemente de su tamaño, dado el riesgo de causar complicaciones letales^3^^,^^11^. La cirugía mínimamente invasiva ha permitido conseguir una recuperación funcional de los pacientes cada vez más precoz^14^. En general, las masas cardíacas tienen buen pronóstico^8^^,^^11^ y un riesgo de recidiva reducido, siendo del 2% en las series más extensas^15^.

La escasa prevalencia y sintomatología inespecífica dificultan la inclusión de las masas cardíacas en el diagnóstico diferencial clínico. Además, la literatura se centra primordialmente en el mixoma y son escasos los datos relativos a otras masas cardíacas menos habituales. En consecuencia, el objetivo de este estudio fue realizar una caracterización exhaustiva de los cuadros presentados por los pacientes diagnosticados de masas y quistes cardíacos en nuestro medio, con el fin de orientar futuros diagnósticos y poder brindarles una óptima atención clínica.

## MATERIAL Y MÉTODOS

Estudio descriptivo, retrospectivo y unicéntrico. Se realizó una búsqueda en la base de datos del Servicio de Cardiología y Cirugía Cardíaca de la Clínica Universidad de Navarra (Pamplona, España) para identificar aquellos pacientes que fueron intervenidos por *tumores y quistes cardíacos* entre diciembre de 2002 y diciembre de 2022.

Se consultaron las historias clínicas para recoger las siguientes variables relacionadas con el proceso descrito:

- sociodemográficas: año de diagnóstico, sexo y edad al diagnóstico;
- clínicas: factores de riesgo cardiovascular (FRCV) como índice de masa corporal (IMC, kg/m^2^) categorizado en normopeso (18,50-24,99), sobrepeso (25,00-29,99), obesidad tipo I (30,00-34,99) y obesidad tipo II (35,00-39,99), hipertensión arterial, diabetes mellitus, dislipemia, hiperuricemia y hábito tabáquico (no fumador, exfumador, fumador activo); antecedentes personales (enfermedad cardiovascular, respiratoria, oncológica, cirugía cardíaca previa) y antecedentes familiares;
- cardiológicas: sintomatología, prueba de imagen de diagnóstico, tipo de hallazgo (incidental, por síntomas), localización anatómica;
- prequirúrgicas: diámetro medio prequirúrgico (mm), prueba de imagen empleada para su medición y diámetro medio medido tras su extirpación (mm);
- anatomopatológicas: tipo de masa cardíaca (mixoma, leiomiosarcoma, sarcoma intimal, quiste intrapericárdico, trombo).

Asimismo, se consultó el informe anatomopatológico y la descripción de las intervenciones quirúrgicas anotada por el equipo quirúrgico.

Tanto en la primera revisión postcirugía (durante el ingreso hospitalario) como en la primera revisión ambulatoria tras el alta hospitalaria se recogió el momento concreto de la revisión (en días postcirugía), sintomatología, pruebas de imagen empleadas y datos de recidiva y muerte.

Este estudio se llevó a cabo en concordancia con la Declaración de Helsinki y fue aprobado por el Comité de Ética de la Investigación de la Universidad de Navarra (proyecto 2023.140). La información referente a pacientes se codificó de forma anonimizada, impidiendo que permitiese identificar a la persona.

*Análisis estadístico*. La normalidad de las variables continuas se exploró mediante la prueba de Shapiro-Wilk y de la asimetría y la curtosis. Las variables con distribución normal se describieron con medias aritméticas (^-^x) y desviaciones estándar (DE) y, en caso contrario, con medianas (P_50_) y rangos intercuartílicos (RIC). Las variables cualitativas se describieron mediante frecuencias y porcentajes. Se empleó la prueba *U* de Mann-Whitney para comparar el diámetro medio postcirugía de las masas halladas en las cámaras cardíacas de acuerdo con su localización anatómica. La concordancia entre los diámetros medios pre y postcirugía se evaluó mediante el coeficiente de correlación intraclase (CCI) junto con su intervalo de confianza del 95% (IC95%). Los valores *p* a dos colas <0,05 se consideraron estadísticamente significativos. Todos los análisis se realizaron con STATA v.15.1 (Stata Corp, College Station, TX, USA) y GraphPad Prism v.9.5.1 (GraphPad Software Inc., San Diego, CA, USA).

## RESULTADOS

Se identificaron 13 pacientes en la búsqueda, que se incluyeron en su totalidad. En la muestra había un predominio de sexo femenino (76,92%) y la edad media fue de 63,08 años (rango: 30-86). Las características basales de los pacientes seleccionados se presentan en la Tabla 1, según el tipo de masa cardíaca diagnosticada.

### Año de diagnóstico

La incidencia acumulada no resultó homogénea a lo largo del periodo de estudio: en el punto medio del estudio (diciembre de 2012) se habían diagnosticado cinco casos (38,56%), y casi la mitad de los casos (53,85%, *n* = 7) fueron diagnosticados en los primeros dieciséis años (hasta diciembre de 2018).

**Figura 1:**
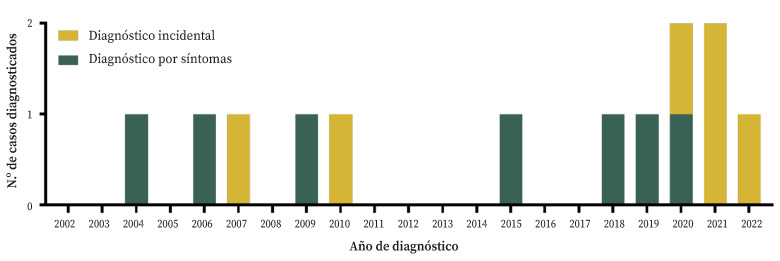
Distribución de los casos diagnosticados a lo largo del tiempo según el tipo de hallazgo.

La incidencia acumulada de diagnósticos incidentales también aumentó en los últimos años: en los primeros diez años supusieron el 28,57% de los diagnósticos frente al 66,66% de los siguientes diez (y el 100% en los dos últimos años) (Fig. 1).

**Tabla 1:** Características basales de los pacientes seleccionados

Variables	Tipo de masa cardíaca					Total
	Mixoma	Leiomiosarcoma	Sarcoma intimal con trombosis asociada	Quiste intrapericárdico	Trombo	
	*n* = 9	*n* = 1	*n* = 1	*n* = 1	*n* = 1	*n* = 13
Sexo (mujer), *n* (%)	8 (88,89)	1 (11,11)	0	1 (11,11)	0	10 (76,92)
Edad (años), *media (DE)*	63,22 (7,48)	64	74	64	49	63,08 (15,17)
Factores de riesgo cardiovascular, *n* (%)						
IMC (kg/m^2^), *media (DE)*	26,36 (4,23)	26,5	30,1	36	24,9	27,36 (5,50)
HTA	5 (55,56)	0	1 (100)	1 (100)	0	7 (53,85)
DM	0	0	1 (100)	1 (100)	2 (15,38)
Dislipemia	5 (55,56)	1 (100)	0	0	0	6 (46,15)
Hiperuricemia	2 (22,22)	0	0	0	0	2 (15,38)
Hábito tabáquico						
No fumador	6 (66,67)	1 (100)	1 (100)	1 (100)	0	9 (69,23)
Exfumador	2 (22,22)	0	0	0	1 (100)	3 (23,08)
Fumador activo	1 (11,11)	0	0	0	0	1 (7,69)
Antecedentes personales, *n* (%)						
Cardiovasculares	2 (22,22)	0	0	0	1 (100)	3 (23,08)
Respiratorios	1 (11,11)	0	1 (100)	1 (100)	0	3 (23,08)
Oncológicos	2 (22,22)	0	0	0	0	2 (15,38)
Cirugía cardíaca previa	0	0	0	0	1 (100)	1 (7,69)
Antecedentes familiares, *n* (%)						
Cardiovasculares	2 (22,22)	0	0	0	1 (100)	3 (23,08)
Sintomatología, *n* (%)						
Asintomático	3 (33,33)	0	0	0	1 (100)	4 (30,77)
Disnea	4 (44,44)	1 (100)	1 (100)	1 (100)	0	7 (53,85)
Palpitaciones en reposo	2 (22,22)	0	0	0	0	2 (15,38)
Malestar general	1 (11,11)	0	0	0	0	1 (7,69)
Astenia	2 (22,22)	0	0	0	0	2 (15,38)
Episodios presincopales	2 (22,22)	0	0	0	0	2 (15,38)
Dolor epigástrico	1 (11,11)	0	0	0	0	1 (7,69)
Dolor torácico no relacionado con el esfuerzo	1 (11,11)	0	0	0	0	1 (7,69)
Edemas EEII	1 (11,11)	0	0	1 (100)	0	2 (15,38)
Edemas EESS	1 (11,11)	0	0	0	0	1 (7,69)
Prueba de imagen de diagnóstico, *n* (%)						
ETT Doppler color	8 (88,89)	0	0	0	1 (100)	9 (69,23)
AngioTC de tórax	0	0	1 (100)	0	0	1 (7,69)
TC de tórax	1 (11,11)	0	0	0	0	1 (7,69)
TC de tórax con contraste iv	0	1 (100)	0	1 (100)	0	2 (15,38)
Tipo de hallazgo, *n* (%)						
Incidental	4 (44,44)	0	0	1 (100)	1 (100)	6 (46,15)
Por clínica	5 (55,56)	1 (100)	1 (100)	0	0	7 (53,85)
Localización anatómica, *n* (%)						
Aurícula derecha	1 (11,11)	0	0	0	1 (100)	2 (15,38)
Aurícula izquierda	8 (88,89)	1 (100)	0	0	0	9 (69,23)
Predominancia derecha	0	0	0	1 (100)	0	1 (7,69)
Tronco pulmonar	0	0	1 (100)	0	0	1 (7,69)
Diámetro medio precirugía (mm), *media (DE)*	34,31 (13,85)	*	37	79	28,5	37,78 (17,65)
Técnica de medición, *n* (%)						
ETT Doppler color	6 (66,67)	1 (100)	0	0	1 (100)	8 (61,54)
ETE Doppler color	2 (22,22)	0	0	0	0	2 (15,38)
AngioTC de tórax	0	0	1 (100)	0	0	1 (7,69)
TC de tórax con contraste iv	0	0	0	1 (100)	0	1 (7,69)
RMN de corazón	1 (11,11)	0	0	0	0	1 (7,69)
Diámetro medio postcirugía (mm), *media (DE)*	38,00 (17,15)	**	40	***	20	36,55 (16,31)

AngioTC: angiografía por tomografía computarizada; DE: desviación estándar; DM: diabetes mellitus; EEII: extremidades inferiores; EESS: extremidades superiores; ETE: ecocardiograma transesofágico; ETT: ecocardiograma transtorácico; HTA: hipertensión arterial; IMC: índice de masa corporal; iv: intravenoso; RMN: resonancia magnética nuclear; TC: tomografía computarizada; *: no medido; **: masa muy infiltrante, imposible de escindir unitariamente y medir con exactitud; ***: no medido por tratarse de una colección líquida.

### Tipo de masa cardíaca y localización anatómica

El tipo de masa cardíaca más frecuente fue el mixoma (69,23%, *n* = 9), la mayoría (88,89%, *n* = 8) localizados en la aurícula izquierda, y solo uno en la derecha. En un caso la masa se hallaba implantada en la fosa oval, protruyendo al ventrículo izquierdo en diástole y condicionando una regurgitación mitral. El informe histológico recogía que, además del fondo de mucopolisacáridos que presentaban todos ellos, dos casos contenían focos de necrosis hemorrágica, uno asociaba colonias bacterianas y otro poseía un notable componente vascular con vasos mal formados.

Un paciente presentó un leiomiosarcoma en la aurícula izquierda, aunque ocupaba completamente la luz de ambas venas pulmonares derechas y se extendía hasta las lobares e incluso hasta las venas segmentarias. El equipo quirúrgico evidenció que se trataba de un tumor blanquecino muy duro e infiltrante, lo que imposibilitó su medición e incluso la escisión en un único fragmento. El informe histológico indicó que la masa presentaba una gran heterogeneidad, con microcalcificaciones y abundante infiltrado inflamatorio.

Un paciente fue diagnosticado de sarcoma maligno de la íntima con trombosis asociada. El tumor se hallaba en el tronco pulmonar, extendiéndose hacia la arteria pulmonar derecha y en menor medida hacia la izquierda. La masa presentaba una diferenciación muscular y el estudio de hibridación fluorescente *in situ* (FISH) reveló una amplificación del gen *mdm2.* Tras la resección se ofreció tratamiento adyuvante consistente en radioterapia (técnica IMRT/VMAT) y quimioterapia (temozolomida), pero el paciente no se trató en el centro.

En otro paciente se halló un quiste intrapericárdico extracavitario de predominancia derecha, de forma que comprimía la aurícula y el ventrículo derechos, desplazando anteriormente la arteria coronaria derecha e incluso prolongándose por la cara inferior del corazón. En un primer momento se planteó un diagnóstico diferencial entre derrame pericárdico encapsulado y hemopericardio de evolución crónica. Al drenar la colección líquida, se vio que contenía un fondo de hematíes parcialmente lisados y abundante contenido necrótico correspondiente a una necrosis de coagulación, sin observarse infiltrado inflamatorio ni células viables, por lo que se llegó a la conclusión de que la colección correspondía a un hemopericardio antiguo.

Por último, un paciente presentó un trombo de fibrina en la aurícula derecha anclado en la red de Chari.

### Factores de riesgo cardiovascular

Solo un paciente (7,69%) no presentaba FRCV, el 69,23% (*n* = 9) presentaron dos, más de la mitad (53,85%, *n =* 7), tres, y un paciente presentó hasta cuatro FRCV.

**Figura 2:**
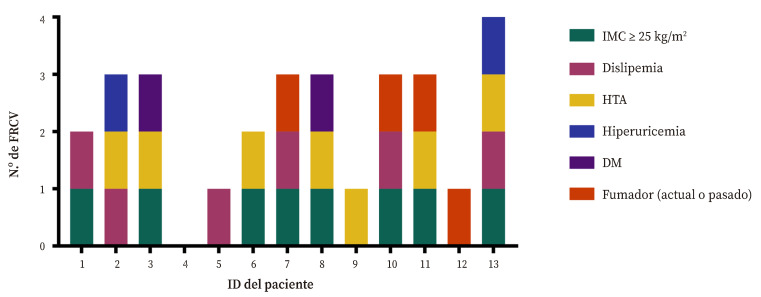
Relación de factores de riesgo cardiovascular presentados por cada paciente.

DM: diabetes mellitus; FRCV: factores de riesgo cardiovascular; HTA: hipertensión arterial; IMC: índice de masa corporal.

El sobrepeso/obesidad fue el FRCV más prevalente (61,54%, *n* = 8), con cinco pacientes con sobrepeso (38,46%), uno con obesidad tipo I (7,69%) y dos con obesidad tipo II (15,38%). Le siguieron la hipertensión arterial (53,85%, *n* = 7) y la dislipemia (46,15%, *n* = 6) (Fig. 2).

### Antecedentes personales y familiares

Tres pacientes presentaban un antecedente de enfermedad respiratoria (asma bronquial, tuberculosis pulmonar, síndrome de apnea-hipopnea obstructiva del sueño), dos presentaban antecedentes cardiovasculares (fibrilación auricular, arteritis de Horton y síndrome varicoso bilateral) y otros dos antecedentes oncológicos (carcinoma basocelular en la nariz, macroadenoma hipofisario infiltrante secundario a una acromegalia). Un paciente ya había sido previamente intervenido por el equipo de Cirugía Cardíaca por el cierre de una comunicación interauricular tipo *ostium secundum*, sufriendo un ictus en el postoperatorio. Sin embargo, este proceso tuvo lugar tres años antes del diagnóstico de la masa cardíaca y la masa detectada, un trombo de fibrina, no se hallaba en contacto con ese cierre de la comunicación interauricular previa.

Respecto a los antecedentes familiares relevantes, la abuela materna de un paciente padecía una arritmia, y la madre de otro paciente tenía diabetes mellitus y su padre, diabetes mellitus e hipertensión arterial. La madre de un tercer paciente tenía hipertensión arterial y diabetes mellitus, y había sufrido un infarto agudo de miocardio; su padre, un accidente cerebrovascular y un infarto de miocardio, y tres de sus hermanos, un ictus. Ningún paciente refirió que tuviese familiares que hubiesen estado afectados por masas cardíacas.

### Sintomatología y tipo de hallazgo

El síntoma más prevalente fue la disnea, presente en la mitad de los pacientes (53,85%, *n* = 7), seguida de palpitaciones en reposo, astenia, episodios presincopales y edemas en extremidades inferiores, cada de uno de ellos experimentado por dos pacientes.

En casi la mitad de los casos (46,15%, *n* = 6) el hallazgo de la masa cardíaca fue incidental, al realizar una ecocardiografía transtorácica Doppler color. Cuatro pacientes (30,77%) no presentaban ningún síntoma: en el primer paciente, la masa se descubrió en una revisión del cierre de una comunicación interauricular previa; en el segundo, al estudiar un soplo cardíaco ya conocido detectado durante la revisión de una infección del tracto urinario; en el tercero, al valorar en la consulta de Cardiología una fibrilación auricular e hipertensión arterial previas durante el preoperatorio de una corrección de hernia inguinal; y en el cuarto, al acudir a la consulta de Cardiología por iniciativa propia para realizarse un chequeo. En los otros dos pacientes la masa fue descubierta al realizar una TC de tórax mientras se abordaba otra patología: al ingreso hospitalario por una neumonía por SARS-CoV-2 y ante la sospecha de una descompensación asmática (prueba realizada con administración de contraste intravenoso).

Llama la atención que en dos casos con clínica similar (disnea progresiva y edemas en extremidades inferiores) se realizó, en un primer momento, el diagnóstico de asma, y solo ante la persistencia de la sintomatología y la ausencia de respuesta al tratamiento se consiguió llegar al diagnóstico real de masa cardíaca. Sin embargo, no queda claro si se llegó a descartar la enfermedad concomitante de asma bronquial.

En los siete casos restantes (53,95%) se llegó al diagnóstico de la masa cardíaca como fruto de la investigación dirigida a clarificar la etiología causante de la sintomatología presentada por los pacientes.

### Prueba de imagen de diagnóstico

La prueba de imagen más empleada para llevar a cabo el diagnóstico fue la ecocardiografía transtorácica Doppler color (69,23%, *n* = 9), incluidos los cuatro casos correspondientes a pacientes asintomáticos. En los cinco restantes, esta prueba formó parte del estudio realizado ante la sintomatología referida.

Como ya se ha mencionado, la TC de tórax permitió el diagnóstico de un paciente al ingreso hospitalario por una neumonía por SARS-CoV-2, la TC de tórax con contraste intravenoso diagnosticó un paciente ante la sospecha de una descompensación asmática y se empleó en otro paciente, mientras que una angiografía por TC de tórax permitió realizar el diagnóstico en un último caso.

Independientemente de la técnica empleada para el diagnóstico, el estudio se completó con técnicas adicionales hasta en el 38,46% (*n* = 5) de los casos.

### Mediciones precirugía y postcirugía de las masas cardíacas

En el 61,54% (*n* = 8) de los pacientes, la prueba de imagen utilizada para llevar a cabo el diagnóstico -ecocardiografía transtorácica Doppler color- sirvió para medir la masa previa a la cirugía (Tabla 2), ya que la medición se realizó inmediatamente después de identificar la masa. Los cinco pacientes restantes precisaron otra prueba distinta: en dos casos, la información aportada por ecocardiografía transtorácica se completó con ecocardiografía transesofágica, y en otros dos fue el ecocardiograma transtorácico el que siguió a una TC de tórax (en un caso con contraste intravenoso). Un último paciente requirió una RMN para poder realizar el diagnóstico de mixoma (en la ecografía transtorácica se apreciaba una masa sésil con distintas densidades, con diagnóstico dudoso, mientras que en la RMN se apreció una masa hiperintensa en secuencias T1 y T2, sin captación en secuencias de perfusión y con realce heterogéneo en secuencias de realce tardío, sugestivo de necrosis/fibrosis), por lo que se empleó la RMN para medir la masa.

**Tabla 2:** Comparación de las pruebas de imagen empleadas para realizar el diagnóstico y la medición previa a la cirugía de las masas cardíacas

Paciente	Prueba de imagen	
Diagnóstico	Medición precirugía	
1	TC de tórax con contraste iv	ETT Doppler color
2	ETT Doppler color	ETE Doppler color
3	TC de tórax con contraste iv	TC de tórax con contraste iv
4	ETT Doppler color	ETT Doppler color
5	ETT Doppler color	ETT Doppler color
6	ETT Doppler color	ETT Doppler color
7	ETT Doppler color	ETT Doppler color
8	Angiografía por TC de tórax	Angiografía por TC de tórax
9	TC de tórax	ETT Doppler color
10	ETT Doppler color	ETT Doppler color
11	ETT Doppler color	ETE Doppler color
12	ETT Doppler color	ETT Doppler color
13	ETT Doppler color	RMN de corazón

ETE: ecocardiograma transesofágico; ETT: ecocardiograma transtorácico; iv: intravenoso; RMN: resonancia magnética nuclear, TC: tomografía computarizada.

Todos los pacientes fueron intervenidos mediante esternotomía media, además de la utilización de circulación extracorpórea (a excepción del paciente con quiste pericárdico). El objetivo de las intervenciones fue la exéresis completa de las masas, que fue posible en todos los casos excepto en dos: el paciente que presentó el sarcoma intimal de la arteria pulmonar, y el que presentó un leiomiosarcoma en la aurícula izquierda. Este último paciente falleció intraoperatoriamente.

Se consideró como diámetro postcirugía las mediciones realizadas por el equipo quirúrgico tras la extirpación de las masas, antes de obtener biopsias para remitirlas al Servicio de Anatomía Patológica. La concordancia entre los diámetros medios precirugía y postcirugía resultó muy alta (CCI = 0,807, IC95%: 0,450-0,943). El diámetro medio postcirugía de las masas no difirió significativamente (*p* = 0,237) según su localización (aurícula derecha: mixoma y trombo, o izquierda: mixomas y leiomiosarcoma).

### Primera revisión durante el ingreso y primera revisión ambulatoria

Como ya se ha mencionado, una persona falleció durante la cirugía de exéresis. La intervención cursó sin incidencias y, cuando el equipo quirúrgico se encontraba ya suturando, evidenciaron un desgarro de la aurícula izquierda que no fueron capaces de resolver, ocasionando el fallecimiento por una hemorragia difusa.

Aparte de este luctuoso evento, entre la cirugía y la primera revisión durante el ingreso hospitalario no murió ningún paciente y todos se encontraban asintomáticos. A la hora de la recogida de datos solo se consideraron aquellas pruebas de imagen dirigidas al control de la recidiva, por lo que se descartaron otras pruebas realizadas con el fin de vigilar el postoperatorio, como radiografías de tórax o electrocardiogramas (Tabla 3). Solo en un caso no se empleó la ecocardiografía transtorácica para evidenciar la persistencia de la masa. En el resto de los pacientes (91,67%, *n* = 11) se observó que la exéresis había resultado exitosa. Al paciente cuyo diagnóstico había sido sarcoma intimal con trombosis asociada, además de ecocardiografía también se realizó angiografía por TC de tórax (ya que el tumor se hallaba en el tronco pulmonar, extendiéndose hacia ambas arterias pulmonares), en la que se vio cómo el tronco pulmonar y la arteria pulmonar izquierda se encontraban libres de enfermedad, pero persistía un pequeño defecto de repleción que enlentecía el flujo de la arteria lobar superior derecha. Esta fue una de las razones por las que se le ofreció tratamiento adyuvante con radioterapia y quimioterapia tras la cirugía.

**Tabla 3:** Datos recogidos referentes a la primera revisión durante el ingreso hospitalario y a la primera revisión ambulatoria tras el alta hospitalaria

Hospitalaria	Primera revisión	
	Ambulatoria	
	(durante el ingreso)	(tras el alta)
Días postcirugía, *media (DE)*/*P*_*50*_ *(RIC)*	4,83 (2,17)	34 (30-41)
Sin sintomatología, *n* (%)	12 (100)	12 (100)
Prueba de imagen empleada para el control de la recidiva, *n* (%)		
ETT Doppler color	11 (91,67)	-
AngioTC de tórax	1 (8,33)	-
Pruebas complementarias empleadas para el control ambulatorio, *n* (%)		
ETT Doppler color	-	7 (58,33)
ECG	-	7 (58,33)
Radiografía simple de tórax PA y L	-	9 (75,00)
Recidiva, *n* (%)	0	1 (8,33)
Muerte, *n* (%)	1 (7,69)^*^	0

AngioTC: angiografía por tomografía computarizada; DE: desviación estándar; ECG: electrocardiograma; ETT: ecocardiograma transtorácico; L: vista lateral; PA: vista posteroanterior; P_50_: mediana; RIC: rango intercuartílico; -: no aplica; *: frecuencia calculada sobre *n* = 13.

En el momento de la primera revisión ambulatoria tras el alta hospitalaria todos los pacientes se mostraban asintomáticos. La frecuencia de realización de ecocardiograma transtorácico fue menor (58,33%, *n* = 7), y tan solo a cuatro pacientes (33,33%) se les realizaron las tres pruebas (ecocardiograma, electrocardiograma y radiografía de tórax). En diez casos (83,33%) la revisión tuvo lugar un mes después de la cirugía, y en dos, cuatro meses después. Fue precisamente en uno de estos dos casos (cuyo diagnóstico inicial había sido de quiste intrapericárdico) en el que se evidenció una recidiva parcial. A pesar de que el ecocardiograma realizado durante el ingreso no mostraba ninguna alteración, el realizado en este momento mostró una lesión más pequeña que la inicial. Así, existía una masa intrapericárdica en la región lateral de la pared libre del ventrículo derecho, con un diámetro aproximado de 2 cm. Una TC de tórax permitió observar que se trataba de una lesión heterogénea de bordes lobulados y captación periférica de contraste, que se extendía hacia la cara inferior del surco auriculoventricular derecho y hacia el mediastino anterior, hasta el esternón. A pesar de que se recomendó control en dos meses, el paciente no volvió a la consulta. Este fue el único paciente que presentó una recidiva.

## DISCUSIÓN

Este estudio se llevó a cabo con el objetivo de caracterizar exhaustivamente los cuadros presentados por los pacientes diagnosticados de masas y quistes cardíacos en nuestro medio. De hecho, llama la atención que, en la Clínica Universidad de Navarra, un hospital general^16^ que, por sus características, no solo atiende a población navarra sino también a pacientes provenientes de toda España o incluso de ámbito internacional, solo se hayan intervenido 13 casos en los últimos 20 años. Es posible que se detectasen más casos en el centro pero que estos fuesen finalmente operados en los lugares de origen de los pacientes para su mayor comodidad, o en el Servicio de Cirugía Cardíaca del Hospital Universitario de Navarra, hospital general público de referencia para el área de Pamplona e incluso para toda la Comunidad Foral de Navarra en algunos servicios. De cualquier forma, esta cifra responde a la reducida prevalencia de los tumores cardíacos, menor al 0,3%^1^. Por lo tanto, estos datos reflejan el valor que este tipo de estudios presentan a la hora de orientar futuros diagnósticos y comprender el cuadro de estos pacientes, pudiendo así brindarles una óptima atención clínica.

Las masas cardiacas de este estudio presentan unas frecuencias que coinciden con lo publicado, ya que el tumor más observado fue el mixoma (tumor primario benigno más frecuente)^8^ y solo un caso fue diagnosticado de leiomiosarcoma. Aunque el leiomiosarcoma, que deriva de células de músculo liso, afecta comúnmente a órganos abdominopélvicos^19^ como el útero o el intestino, los que afectan al corazón son extremadamente raros y hay pocos casos descritos en la bibliografía^20^. Aunque las metástasis cardíacas, como melanoma, cáncer de mama, pulmón o esófago^17^^,^^18^, son marcadamente más frecuentes que los tumores primarios^5^, ninguno de los pacientes de este estudio presentó una metástasis cardíaca.

Respecto a la localización, el 85% de los mixomas se localizan en la aurícula izquierda^9^, frecuencia que prácticamente coincide con la hallada en este estudio (88,89%). Aunque los sarcomas afectan comúnmente al lado derecho del corazón^3^, en esta serie el paciente lo presentó en la aurícula izquierda.

Los tumores cardíacos deben ser diferenciados de otras masas cardíacas como vegetaciones, trombos o hipertrofia miocárdica^3^, como ocurrió en uno de los pacientes, que presentó un trombo. Un último paciente presentó un sarcoma intimal del tronco pulmonar, un tumor cardiovascular sumamente inusual y agresivo, que normalmente se confunde con un tromboembolismo pulmonar crónico, mucho más frecuente^21^. La invasión local o la metástasis a distancia propia de los sarcomas hacen que el paciente fallezca en meses o incluso semanas^3^.

La media de edad de los pacientes de este estudio (63,08 años) concuerda con que las masas cardíacas aparecen sobre todo entre la tercera y sexta décadas de vida^10^. El 10% de los mixomas son una variante familiar que forma parte del Complejo de Carney, un síndrome neoplásico endocrinológico de transmisión autosómica dominante causado por una mutación en el gen *PRKAR1A*^3^. Mientras que las formas esporádicas suelen ser individuales y aparecer en la aurícula izquierda, adheridas al tabique interauricular, la variante genética puede surgir a edades más tempranas, ser múltiple y localizarse en los ventrículos. En esta serie no hay indicios de que los casos pudiesen tratarse de la variante familiar, salvo en el caso del paciente más joven (30 años), donde al resecar un mixoma de la aurícula izquierda anclado en el septo interauricular se encontró otro mixoma en la aurícula derecha adherido también al septo, de 1 cm de diámetro. Sin embargo, no se cuenta con más información al respecto, como antecedentes familiares de interés.

En la literatura se ha descrito un aumento progresivo de la incidencia de masas cardiacas debido al uso cada vez más extendido de la ecocardiografía como técnica de evaluación del corazón^22^. Además, la mayor calidad y empleo de las técnicas de imagen cardíaca en general también han supuesto un aumento de los descubrimientos incidentales de masas cardíacas^13^. Se ha llegado a estimar que la incidencia de tumores cardíacos primarios se ha doblado en los últimos cincuenta años^23^. Aunque en este estudio llama la atención el aumento de incidencia, no se puede establecer una relación de causalidad con el mayor uso de técnicas de imagen, tanto por la naturaleza retrospectiva del trabajo como por el dato no disponible de la incidencia de casos globales, pues un aumento de incidencia de masas cardíacas operadas debería acompañarse de un aumento concomitante en los diagnósticos.

Algunas series sugieren que los FRCV más prevalentes en estos pacientes son el hábito tabáquico y la dislipemia^24^, mientras que en esta serie fueron el sobrepeso/obesidad y la hipertensión arterial.

Las manifestaciones clínicas son poco específicas y pueden conducir a un diagnóstico erróneo. De hecho, ya se han descrito casos donde el cuadro orientó a un primer diagnóstico de asma bronquial^25^, como ocurrió en esta serie hasta en dos ocasiones. Así, en la literatura se habla con frecuencia de que la presentación clínica típica corresponde a la tríada de obstrucción intracardiaca, tromboembolismo y cuadro constitucional^3^^,^^8^^,^^11^^,^^26^. También es muy común que imiten la enfermedad de válvula mitral, tanto estenosis (por prolapso del tumor en el orifico de la válvula) como insuficiencia (por trauma valvular). El desprendimiento de fragmentos del tumor puede propiciar fenómenos tromboembólicos, y las citoquinas que producen pueden ser la causa de síntomas constitucionales como fiebre, astenia y pérdida de peso^3^^,^^8^.

A pesar de que los pacientes pueden presentar toda esta variedad de síntomas, muchos permanecen asintomáticos en el momento del diagnóstico y este se lleva a cabo de manera incidental a través de estudios de imagen realizados por otra causa^3^, como se observó en esta serie (30,77% asintomáticos y 46,15% diagnósticos incidentales).

Estos estudios de imagen no solo deben estar dirigidos a hacer el diagnóstico diferencial, sino también a evaluar las posibilidades de la cirugía cardíaca^3^^,^^8^. Las guías de cardio-oncología de la Sociedad Europea de Cardiología de 2022^8^ establecen la ecocardiografía como la prueba de elección. Aunque la ecografía transtorácica a menudo resulta suficiente, puede complementarse con la transesofágica, más sensible y específica para detectar tumores más pequeños o situados en localizaciones poco frecuentes^11^^,^^27^^,^^28^. El estudio se puede completar con otras modalidades de imagen como la RMN, útil en la caracterización del tejido de la masa^8^^,^^29^, la TC o la tomografía por emisión de positrones (PET), útiles para distinguir tumores primarios de enfermedad metastásica y tumores benignos de malignos^8^^,^^30^. Sin embargo, la TC no permite diferenciar entre mixomas y trombos^31^, aunque es la mejor para detectar calcificaciones^32^. Por último, en casos señalados, como en pacientes jóvenes que presentan múltiples mixomas, se recomienda el cribado ecocardiográfico de los familiares de primer grado ante la posibilidad de que se trate de un Complejo de Carney^33^.

En la actualidad, el diagnóstico y evaluación preoperatoria se suelen llevar a cabo combinando diferentes técnicas de imagen^3^^,^^7^, como ocurrió en el 38,46% de los casos de esta serie.

A pesar de la vasta caracterización que se puede llevar a cabo con los estudios de imagen, el diagnóstico definitivo debe realizarse mediante el examen histológico de la masa extirpada^11^. Así, de los 265 pacientes diagnósticados de mixoma mediante ecocardiografía en una serie de casos reciente, solo 174 (65,7%) tuvieron un diagnóstico anatomopatológico de mixoma^34^.

Dado su potencial para causar complicaciones graves, el tratamiento de las masas cardíacas es imperativo. Independientemente del tamaño de la masa, la única opción terapéutica es la escisión quirúrgica, cuyo resultado es generalmente curativo^3^^,^^35^^,^^36^. La intervención conlleva un riesgo y un número de complicaciones muy reducidos, siendo los eventos embólicos las más habituales^11^^,^^12^^,^^37^.

Los mixomas resecados tienen muy buen pronóstico^8^^,^^11^, con una supervivencia del 96% a los 10 años^38^. Los tumores malignos, en cambio, presentan un pronóstico pobre; su escisión quirúrgica completa no suele ser posible, por lo que se necesita asociar radioterapia o quimioterapia adyuvantes. En esta serie resultaron incompletas dos resecciones: la del sarcoma intimal del tronco pulmonar y la del leiomiosarcoma en la aurícula izquierda.

La recidiva de las masas cardíacas es muy reducida. Por ejemplo, los mixomas esporádicos recidivan en un 1-2% de los casos (normalmente por una resección incompleta del tumor), si bien los casos familiares lo hacen en un 12-22% (por lesiones multifocales no halladas en un primer momento)^3^. Por eso, se recomienda el seguimiento con ecocardiografía tras la intervención^35^^,^^36^, aunque no queda claro durante cuánto tiempo. En esta serie, en la primera revisión ambulatoria se utilizó ecocardiografía en algo más de la mitad de los pacientes (58,33%).

La principal limitación de este estudio es su carácter retrospectivo que, por ejemplo, impidió la recogida de ciertos datos como el índice paquete-año, el consumo de alcohol y la actividad física practicada por los pacientes. Además, su diseño descriptivo y la inexistencia de un grupo de comparación limita la evaluación de las relaciones causales que se proponen. El diseño unicéntrico acota su validez externa, aunque el hecho de que la Clínica Universidad de Navarra atienda a pacientes de múltiples procedencias nacionales e internacionales palia esta limitación. Por último, debe destacarse también la limitada muestra de masas cardíacas que no sean mixomas. Posibles mejoras para futuros estudios incluyen diseños prospectivos para evitar la pérdida de información, y un carácter multicéntrico con el objetivo de aumentar la validez externa y poder detectar las masas cardíacas menos frecuentes.

En conclusión, esta serie de casos ha permitido caracterizar exhaustivamente los cuadros presentados por los pacientes diagnosticados de masas y quistes cardíacos en nuestro medio, destacando que la práctica totalidad de los pacientes eran mujeres. Se ha conseguido describir un caso de leiomiosarcoma cardíaco y un caso de sarcoma intimal del tronco pulmonar, dos tipos de tumores extremadamente raros de los que existen muy pocos casos descritos en la literatura. También se han detallado otros aspectos poco descritos, como los factores de riesgo cardiovascular más frecuentes en estos pacientes. La descripción de estas patologías tan poco frecuentes y con presentaciones clínicas tan inespecíficas permiten obtener el conocimiento necesario para orientar futuros diagnósticos y brindar una óptima atención clínica a estos pacientes.
